# Anti-depressant-like effects of rannasangpei and its active ingredient crocin-1 on chronic unpredictable mild stress mice

**DOI:** 10.3389/fphar.2023.1143286

**Published:** 2023-03-16

**Authors:** Yang Du, Yan-Li Wang, Lei Chen, Qi-En Li, Yong Cheng

**Affiliations:** ^1^ Key Laboratory of Ethnomedicine of Ministry of Education, Center on Translational Neuroscience, School of Pharmacy, Minzu University of China, Beijing, China; ^2^ Tibetan Medical College, Qinghai University, Xining, Qinghai, China; ^3^ Institute of National Security, Minzu University of China, Beijing, China; ^4^ NHC Key Laboratory of Birth Defect Research, Prevention and Treatment (Hunan Provincial Maternal and Child Healthcare Hospital), Changsha, Hunan, China

**Keywords:** rannasangpei, crocin-1, depression, oxidative stress, inflammatory response

## Abstract

Major depressive disorder is one of the most common neuropsychiatric diseases and it is a global public health problem that leads to disabilities. Currently, there is a growing need to explore novel strategy to cure major depressive disorder due to the limitation of available treatments. Rannasangpei (RSNP) is a traditional Tibetan medicine which acts as a therapeutic agent in various acute or chronic diseases, including cardiovascular diseases and neurodegenerative diseases. Crocin-1 a coloring ingredient of saffron which exhibited anti-oxidative and anti-inflammatory properties. Here, we aimed to illustrate whether RSNP and its active ingredient crocin-1 rescue depressive-like phenotypes in chronic unpredictable mild stress (CUMS) induced mouse model of depression. Our results showed that peripheral administration of RSNP or crocin-1 ameliorated the depressive-like behaviors in CUMS-treated mice, as demonstrated by the forced swimming test and tail suspension test. Furthermore, RSNP or crocin-1 treatment reduced oxidative stress in the peripheral blood and hippocampus of the CUMS-treated mice. Additionally, the dysregulated immune system response, as demonstrated by the increased expression of the pro-inflammatory factors (tumor necrosis factor-α and interleukin-6) and the decreased expression of the anti-inflammatory factor-interleukin-10 in the prefrontal cortex and/or hippocampus of CUMS-treated mice, were at least partially restored by RSNP or crocin-1 treatment. RSNP or crocin-1 also restored apoptotic protein marker (Bcl-2 and Bax) levels in the prefrontal cortex and hippocampus of the CUMS-treated mice. Moreover, our data indicated that RSNP or crocin-1 increased astrocyte number and brain-derived neurotrophic factor levels in the hippocampus of CUMS-treated mice after RSNP or crocin-1 administration. Taken together, our study for the first time revealed an anti-depressant effect of RSNP and its active ingredient crocin-1 in a mouse model of depression, with involvement of oxidative stress, inflammatory response and apoptotic pathway.

## Introduction

Major depressive disorder (MDD) is one of the most common neuropsychiatric diseases, and it is a global public health problem that leads to disabilities (Li et al., 2021). Although the pathogenesis of MDD is complex with involvement of genetics and environmental factors, it is generally considered that stress is a crucial risk factor for the onset and/or development of MDD (Allen and Dwivedi, 2020). Additionally, studies have suggested that stress-induced depression was accompanied by abnormalities in various brain regions, particularly in prefrontal cortex and hippocampus (Savitz and Drevets, 2009; Jiang et al., 2018). Currently, the first-line antidepressants are almost exclusively based on monoamine hypothesis which dominated the pathophysiological role of MDD historically (Davidson, 2010; Fakhoury, 2016). However, it has been reported that approximately half of MDD patients failed to respond to the first-line antidepressants (MacQueen et al., 2017), and these were accompanied by side effects (Khawam et al., 2006). More recently, ketamine has been approved by food and drug administration as a rapid responsive antidepressant, but it also showed intolerant side effects (Wei et al., 2020). Nevertheless, finding novel or alternative treatments with more efficacy and/or less side effects for MDD is an urgent task in the field.

Over the last decade, there is a growing interest to utilize natural products as therapeutic agents for treatment of neurological disorders, such as Alzheimer’s disease, Parkinson’s disease and MDD (Rehman et al., 2019; Dai et al., 2022). These natural products include herbs and food extracted from plants that have been used to treat various human diseases, and it is very populous in different cultures partly due the low economic costs and low risks of side effects (Atanasov et al., 2021). Rannasangpei (RSNP), which is also known as 70 flavors pearl pill, is a traditional Tibetan medicine, that has been widely used in cardiovascular, gastrointestinal, and neurodegenerative diseases, and these were documented in 2015 edition of Pharmacopoeia of China (Nie et al., 2021). As a confidential Tibetan prescription, the precise composition and preparation method of RSNP have not been revealed to the scientists and publics. However, it is known that the active constituents present in RSNP included pearl, Hong-sik, Nine stone, Saffron, Bezoar, Musk and Zuotai, and it has been suggested that saffron is a critical player for the observed effects of RSNP (Nie et al., 2021). Saffron, the dried stigmas of *Crocus sativus* L. is conventionally used as a spice in the world, and it is also used to treat human diseases such as genitourinary complications in different cultures as traditional medicine (Hosseini et al., 2018). Of the compounds extracted from saffron, crocin-1 is well known as a major pharmacological active constituent (Inoue et al., 2018). Additionally, crocin has been reported to be served as a neuroprotective agent to protect against ischemia/reperfusion-induced apoptosis of retinal ganglion cells *via* PI3K/Akt signaling pathway (Qi et al., 2013).

These above results indicate that RSNP and its active ingredients possess neuroprotective properties, and therefore may expand the therapeutic potential into neuropsychiatric diseases. Here, we aimed to test the potential effects of RSNP and its active ingredient crocin-1 on chronic unpredictable mild stress (CUMS) induced-depression in mice. We showed that peripheral administration of RSNP or crocin-1 exhibited anti-depressant-like effects in CUMS-treated mice, we further analyzed oxidative stress status, immune system response, apoptotic protein markers in CUMS-treated mice after RSNP or crocin-1 administration.

## Materials and methods

### Animals

Six-week old male C57BL/6 mice were purchased from Beijing Vital River Laboratory Animal Technology Co. Ltd. (Beijing, China). All the animals were given food and water in a temperature- and humidity-controlled room with a light: dark cycle of 12 h. All animal experiments and protocols were conducted in accordance with the National Institutes of Health Laboratory Animal Care and Use Guidelines (NIH Publication No. 80-23) and were approved by the Animal Care and Use Committee of Minzu University of China.

### CUMS model establishment and behavioral tests

**FIGURE 1:**
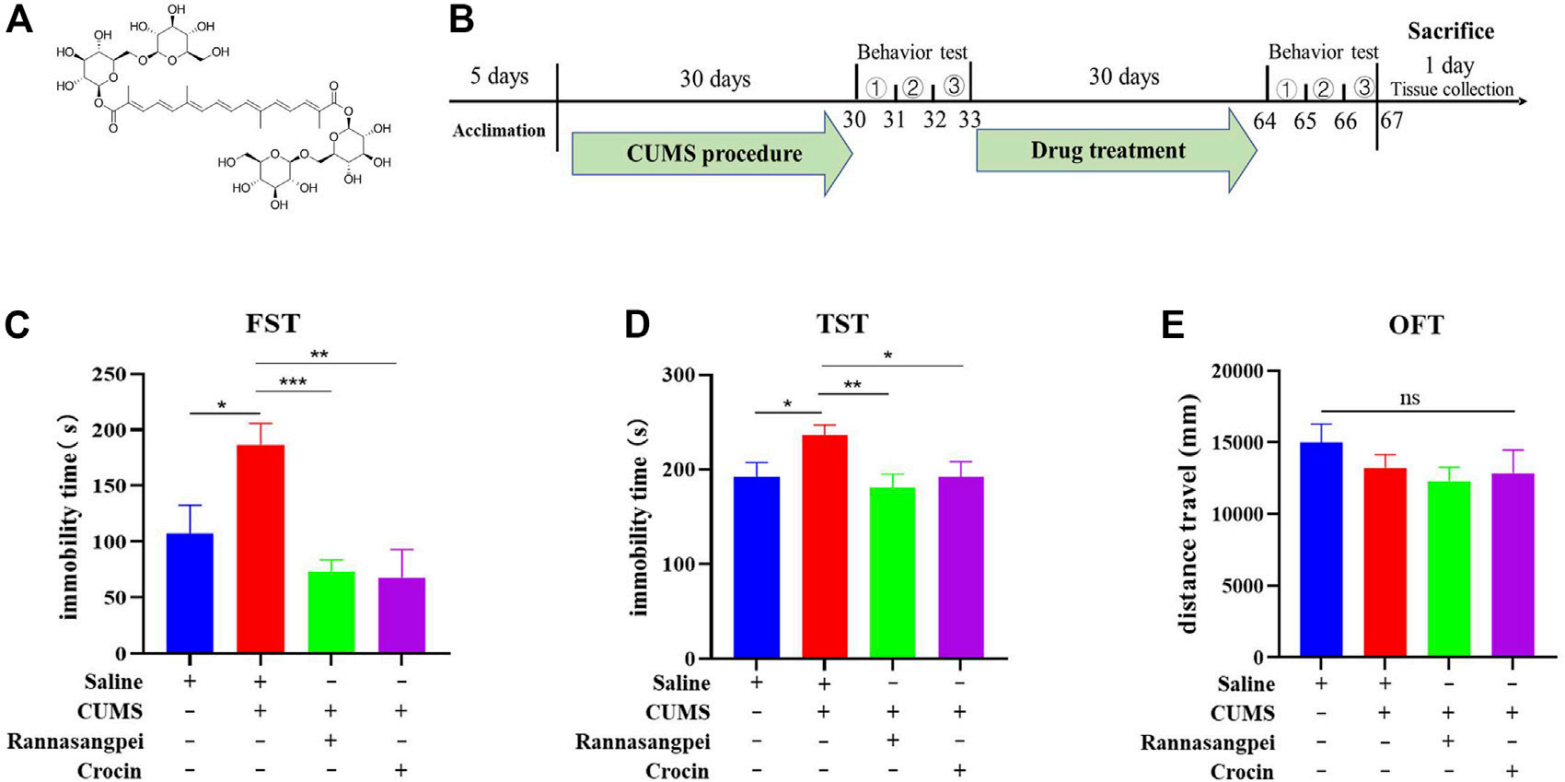
RSNP and crocin-1 rescued stress-induced depressive–like behaviors in mice. **(A)** The molecular structure of crocin-1. **(B)** Schematic representation of the experimental design. RSNP or crocin-1 reduced immobility time in CUMS-treated mice as measured by the FST **(C)** and TST **(D)**. **(E)** The OFT suggested that locomotor activity in mice was not altered by RSNP or crocin-1 treatment (the *y*-axis represents the total distance traveled during the entire testing period). FST, forced swimming test; OFT, open-field test; TST, tail suspension test; RSNP, rannasangpei; CUMS, chronic unpredictable mild stress., represent OFT, TST and FST, respectively. N = 10. For ANOVA statistics: **(C)**, F_(3,40)_ = 7.472, *p* < 0.001; **(D)**, F_(3,40)_ = 3.905, *p* = 0.025; **(E)**, F_(3,40)_ = 0.965, *p* = 0.42; *p < 0.05, **p < 0.01, ***p < 0.001.

After 5-day acclimation, the mice were subjected to CUMS to induce depressive-like behaviors according to our previous publication (Wang et al., 2018). The mice were randomly divided into four groups: control group, CUMS model group, CUMS model plus RSNP group, CUMS model plus crocin-1 group. After 30 days of CUMS treatment, the mice were intragastrically administered with RSNP (1 g/kg, diluted in saline) or crocin-1 (molecular structure see Figure 1A, 5 mg/kg) for 30 days (Figure 1B). It should be noted that the dose selection of RSNP in this study was based on the dose prescribed for humans. We used behavioral tests to evaluate whether RSNP and crocin-1 had anti-depressant-like effects. The day after the behavioral tests, all the mice were sacrificed and their blood and brain tissues were collected for biochemical assays. The experimental procedure is shown in Figure 1B. Behavioral tests used in this study included forced swimming test (FST), open-field test (OFT) and tail suspension test (TST) which were performed as described in our recent publication (Wang et al., 2018). Briefly, for OFT, each mouse was carefully placed in the center of the open-field apparatus (50 cm × 50 cm × 45 cm, Chengdu Taimeng Software Co. Ltd., Chengdu, China). The movement of each moue was recorded for 5 min by a video camera, and the data were analyzed using an open-field software (Chengdu Taimeng Software Co. Ltd.). For FST, mice were placed in a glass cylinder (Chengdu Taimeng Software Co. Ltd.) filled with water (approximately 25°C) for a period of 6 min. After the first 1 min of adaption, the immobility time of the mouse in the last 5 min was recorded and analyzed by the FST software (Chengdu Taimeng Software Co. Ltd.). For TST, the mouse was individually suspended upside down in the tail suspension chamber (Chengdu Taimeng Software Co. Ltd.). After 1 min of adaptation, TST software was used to record and analyze the immobility time of the mice over the subsequent 5 min.

### Measurement of oxidative stress markers

Sample preparation for oxidative stress markers on hippocampal tissues was performed as previously described (Wang et al., 2018). Briefly, hippocampal tissues were lysed and the homogenates were centrifuged to collect supernatants. The protein concentration in supernatants was subsequently measured using the BCA protein assay kit (Solarbio, Beijing, China). Then, the samples from hippocampus and serum were used to investigate oxidative stress marker levels in the mice after various treatments. Superoxide dismutase (SOD), malondialdehyde (MDA), nitric oxide (NO), total antioxidant capacity (T-AOC) and catalase activities or levels were measured by the relative kits according to the instructions provided by the manufacturer (Nanjing Jiancheng Bioengineering Institute, Nanjing, China).

### Quantitative real-time PCR (qRT-PCR)

Transcriptional expression levels of cytokines (IL-6, IL-10 and TNF-α) and apoptosis-related markers (Bax and Bcl-2) were examined by qRT-PCR according to our previous literature (Du et al., 2020). Briefly, hippocampal and prefrontal cortical tissues were dissected from mice under various treatments, and total RNAs in the samples were extracted by trizol reagent (ThermoFisher Scientific, Waltham, MA, US). Then RNAs were reverse-transcribed into cDNAs using StarScript II RT Mix with gDNA Remover (GenStar, Beijing, China). PCR amplification was carried out in the presence of cDNA template, SYBR green I Master Mix (Solarbio, Beijing, China) and forward and reverse primers, on a LightCycler^®^ 96 system (Roche, Basel, Switzerland). The cycling conditions were: 10 min denaturation at 95°C and 40 cycles of DNA synthesis at 95°C for 15 s and 60°C for 1 min. The primer sequences were shown in Table 1. The relative transcriptional expression levels were analyzed using the 2^−ΔΔCT^ method. It should be noted that the coordinates of the brain regions analyzed according to Bregma ATLAS were: prefrontal cortex (Interaural: 6.88 mm–5.14 mm; Bregma:3.08 mm–1.34 mm, lateral: 1.25 mm–3 mm); Hippocampus (Interaural: 2.68 mm to −0.24 mm; Bregma: 0.94 mm to −4.04 mm, lateral:2 mm–4.25 mm).

**TABLE 1:** Primer sequences for qRT-PCR.

Primers	Forward primer	Reverse primer
m-IL-6	TGG​CTA​AGG​ACC​AAG​ACC​ATC​CAA	AAC​GCA​CTA​GGT​TTG​CCG​AGT​AGA
m-IL-10	CCA​AGG​TGT​CTA​CAA​GGC​CA	GCT​CTG​TCT​AGG​TCC​TGG​AGT
m-TNF-α	GCT​CCT​CCA​CTT​GGT​GGT​TTG​T	ACT​CCA​GGC​GGT​GCC​TAT​GTC
m-Bax	TGC​TAG​CAA​ACT​GGT​GCT​CA	CTT​GGA​TCC​AGA​CAA​GCA​GC
m-Bcl2	GGC​CTT​CTT​TGA​GTT​CGG​TG	GCA​TGC​TGG​GGC​CAT​ATA​GTT
m-Actin	AGA​CCT​CTA​TGC​CAA​CAC​AGT	TCC​TGC​TTG​CTG​ATC​CAC​AT

Note: qRT-PCR, Quantitative real-time Polymerase Chain Reaction; IL-6, interleukin six; IL-10, interleukin 10; TNF-α, tumour necrosis factor alpha-like; Bax, BCL2-associated X protein; Bcl 2, BCL2 apoptosis regulator.

### Western blotting

Western blot analysis was performed as described previously (Du et al., 2019). Briefly, 30 μg proteins in the lysate samples from hippocampal and prefrontal cortical tissues were separated by 10% SDS-PAGE for 1.5 h and then transferred to a 0.22 μm polyvinylidene fluoride membrane (ThermoFisher Scientific). After membrane blocking, it was incubated with a primary antibody at 4°C overnight, followed with three-time washing. The membrane was then incubated with a horseradish peroxidase-conjugated secondary antibody (Jackson ImmunoResearch Laboratories Inc., PA, United States). After washing, the signals were visualized using a chemiluminescence detection system (Tanon 4200, Shanghai, China), and the images were quantified using the ImageJ software. Monoclonal mouse BDNF (1:1000) and β-Actin (1:10000) antibodies were purchased from Abcam (Cambridge, United Kingdom) and Cell Signaling Technology (Boston, MA, United States), respectively. Secondary goat anti-mouse and goat anti-rabbit antibodies were purchased from Abcam.

### Immunohistochemisty

Given it has been suggested that BDNF prevented astrocyte death resulting in neuroprotection (Saba et al., 2018), we immunostained glial fibrillary acidic protein (GFAP) in the hippocampus to analyze astrocyte numbers in hippocampus of mice under various treatments by immunohistochemistry as described previously (Cheng et al., 2015). Mon + oclonal mouse anti-GFAP antibody (1:1000) was from Cell Signaling Technology. The Alexa Fluor 488 goat anti-mouse secondary antibody (1:1000) was from ThermoFisher Scientific.

### Statistical analysis

All data analyses were conducted using the GraphPad Prism 8.0 software. Results were expressed as the mean ± SEM (standard error of the mean). Statistical significance was calculated using one-way analysis of variance (ANOVA) followed by multiple comparison tests. *p* values less than 0.05 (*p* < 0.05) were considered statistically significant.

## Results

### RSNP and crocin-1 rescued depressive-like behaviors in CUMS-treated mice

To assess the potential effects of RSNP and crocin-1 in depression, we intragastrically administered RSNP and crocin-1 into the CUMS-treated mice, and used FST, TST and OFT to evaluate the depressive-like behaviors. The results from FST showed that the CUMS-treated mice had higher levels of immobility than the saline-treated mice. However, RSNP or crocin-1 administration significantly reduced immobility time in the CUMS-treated mice, suggesting the anti-depressant-like effects of RSNP and crocin-1 (Figure 1C). To further confirm the rescue effects of RSNP and crocin-1 in depression, we performed TST and demonstrated that RSNP or crocin-1 also significantly decreased immobility time in the CUMS-treated mice (Figure 1D). Additionally, OFT showed that RSNP or crocin-1 treatment did not significantly alter locomotor activity in the CUMS-treated mice (Figure 1E), suggesting that the effects of RSNP and crocin-1 were specific for depressive-like behaviors and were not due to a general increase in motor activity. These results demonstrated that RSNP and its active ingredient crocin-1 rescued depressive-like behaviors in the CUMS-treated mice.

### RSNP and crocin-1 reduced oxidative stress in CUMS-treated mice

**FIGURE 2:**
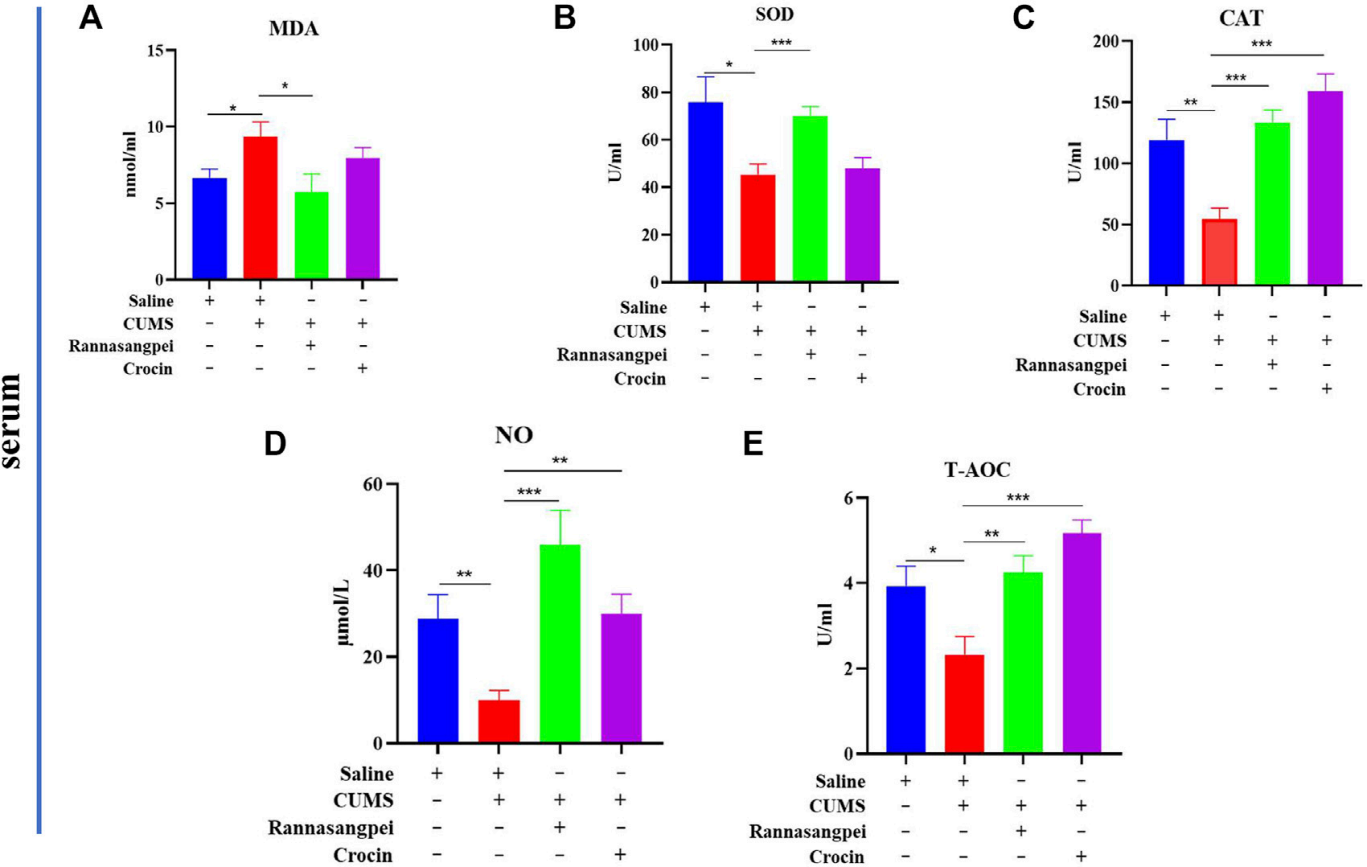
RSNP and crocin-1 restored oxidative stress marker levels in serum of CUMS-treated mice **(A)** The CUMS-treated mice exhibited elevated serum MDA levels, whereas RSNP, but not crocin-1 administration significantly reduced MDA levels in the CUMS-treated mice. **(B)** The decreased SOD activity found in the CUMS-treated mice was restored by RSNP, but not crocin-1 administration. RSNP and crocin-1 significantly increased CAT **(C)**, NO **(D)** and T-AOC **(E)** activities or levels in the CUMS-treated mice. SOD, superoxide dismutase; MDA, malondialdehyde; CAT, catalase; NO, nitric oxide; T-AOC, total antioxidant capacity; RSNP, rannasangpei; CUMS, chronic unpredictable mild stress. N = 8. For ANOVA statistics: **(A)**, F_(3,32)_ = 3.45, *p* = 0.032; **(B)**, F_(3,32)_ = 5.723, *p* = 0.0031; **(C)**, F_(3,32)_ = 11.47, *p* < 0.001; **(D)**, F_(3,32)_ = 5.136, *p* = 0.0057; **(E)**, F_(3,32)_ = 8.867, *p* < 0.001. *p < 0.05, **p < 0.01, ***p < 0.001.

To evaluate the potential mechanisms underlying the anti-depressant-like effects of RSNP and crocin-1, oxidative stress markers in mice under various treatments were measured. The results showed that compared to the saline-treated mice, the CUMS-treated mice had significantly increased serum MDA levels. Interestingly, we found that RSNP, but not crocin-1 administration significantly reduced serum MDA levels in the CUMS-treated mice (Figure 2A). Consistently, the decreased serum SOD activities found in the CUMS-treated mice were significantly rescued by the treatment of RSNP, but not crocin-1 (Figure 2B). In contrast, our results indicated that catalase (Figure 2C), NO (Figure 2D) and T-AOC (Figure 2E) activities or levels in the CUMS-treated mice were lower than that of saline-treated mice, and both RSNP and crocin-1 significantly increased catalase, NO and T-AOC activities or levels in the CUMS-treated mice (Figures 2C–E).

**FIGURE 3:**
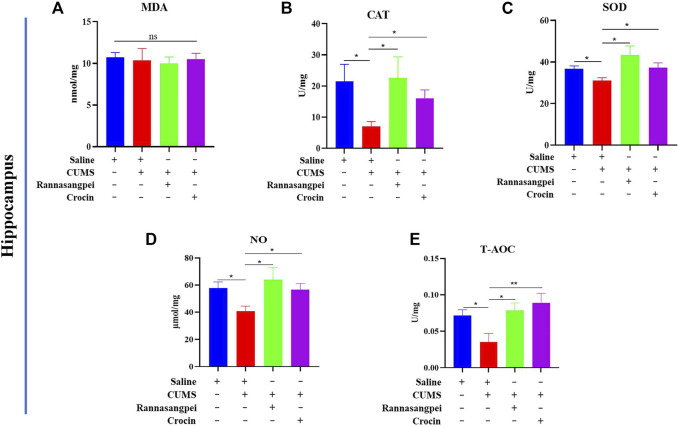
RSNP and crocin-1 restored oxidative stress marker levels in hippocampus of CUMS-treated mice **(A)** Hippocampal MDA levels did not show significant differences among groups. RSNP and crocin-1 significantly increased hippocampal CAT **(B)**, SOD **(C)**, NO **(D)** and T-AOC **(E)** activities or levels in the CUMS-treated mice. SOD, superoxide dismutase; MDA, malondialdehyde; CAT, catalase; NO, nitric oxide; T-AOC, total antioxidant capacity; RSNP, rannasangpei; CUMS, chronic unpredictable mild stress. N = 7. For ANOVA statistics: **(A)**, F_(3,28)_ = 0.1201, *p* = 0.947; **(B)**, F_(3,28)_ = 3.39, *p* = 0.042; **(C)**, F_(3,28)_ = 3.425, *p* = 0.033; **(D)**, F_(3,28)_ = 4.523, *p* = 0.017; **(E)**, F_(3,28)_ = 4.604, *p* = 0.011.*p < 0.05, **p < 0.01, ***p < 0.001.

We further analyzed oxidative stress marker levels in the hippocampus of mice under various treatments. In contrast to the findings in serum, hippocampal MDA levels did not show significant differences among groups (Figure 3A). However, catalase, SOD, NO and T-AOC activities or levels were significantly reduced in the hippocampus of when compared with saline-treated mice, and RSNP or crocin-1 administration significantly increased hippocampal catalase, SOD, NO and T-AOC activities or levels in the CUMS-treated mice (Figures 3B–E).

These results demonstrated that both RSNP and crocin-1 had strong antioxidant effects in the stressed mice, although RSNP showed more potent effects than crocin-1.

### RSNP and crocin-1 had anti-inflammatory and anti-apoptotic effects in CUMS-treated mice

**FIGURE 4:**
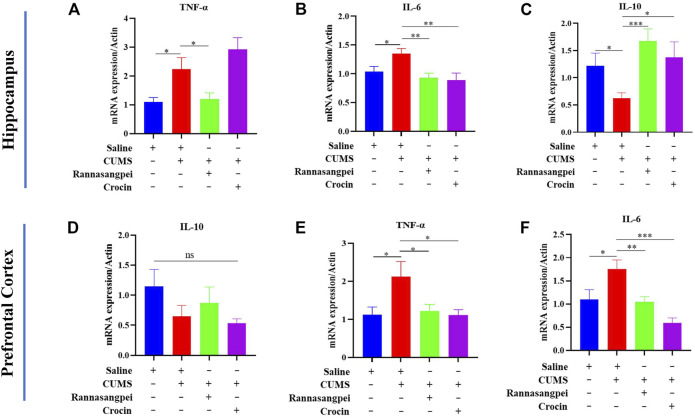
RSNP and crocin-1 inhibited inflammatory response in the brains of CUMS-treated mice **(A)** RSNP, but not crocin-1 inhibited CUMS-induced upregulation of TNF-α transcriptional expression in the hippocampus. **(B)** Both RSNP and crocin-1 inhibited CUMS-induced upregulation of IL-6 transcriptional expression in the hippocampus. **(C)** Both RSNP and crocin-1 restored CUMS-induced downregulation of IL-10 transcriptional expression in the hippocampus. **(D)** IL-10 transcriptional expression levels did not show significant differences among groups in the prefrontal cortex. RSNP and crocin-1 inhibited CUMS-induced upregulation of TNF-α **(E)** and IL-6 **(F)** transcriptional expression in the prefrontal cortex. It should be noted that all the data were normalized to the saline group. TNF, tumor necrosis factor; IL, interleukin; RSNP, rannasangpei; CUMS, chronic unpredictable mild stress. N = 9. For ANOVA statistics: **(A)**, F_(3,36)_ = 8.050, *p* < 0.001; **(B)**, F_(3,36)_ = 4.283, *p* = 0.012; **(C)**, F_(3,36)_ = 3.833, *p* = 0.018; **(D)**, F_(3,36)_ = 1.280, *p* = 0.306; **(E)**, F_(3,36)_ = 3.885, *p* = 0.023; **(F)**, F_(3,36)_ = 8.922, *p* < 0.001. *p < 0.05, **p < 0.01, ***p < 0.001.

Next, we analyzed cytokine and apoptosis-related marker expressions in mice under various treatments. The results showed TNF-α and IL-6 (pro-inflammatory genes) transcriptional expression levels were significantly increased in the hippocampus of CUMS-treated mice compared with saline-treated mice, and RSNP administration reduced the TNF-α and IL-6 transcriptional levels in the CUMS-treated mice (Figures 4A, B). Noticeably, crocin-1 reduced the IL-6, but not TNF-α transcriptional expression levels in the hippocampus of CUMS-treated mice (Figures 4A, B). Furthermore, the data indicated that the reduced IL-10 (anti-inflammatory gene) mRNA levels in the hippocampus of CUMS-treated mice were rescued by RSNP or crocin-1 administration (Figure 4C). In the prefrontal cortex, we did not find significant differences among groups evaluating IL-10 mRNA levels (Figure 4D). However, we found that TNF-α and IL-6 mRNA levels were significantly downregulated in the prefrontal cortex of CUMS-treated mice compared to the saline-treated mice, whereas RSNP or crocin-1 rescued the observed effects (Figures 4E, F).

**FIGURE 5:**
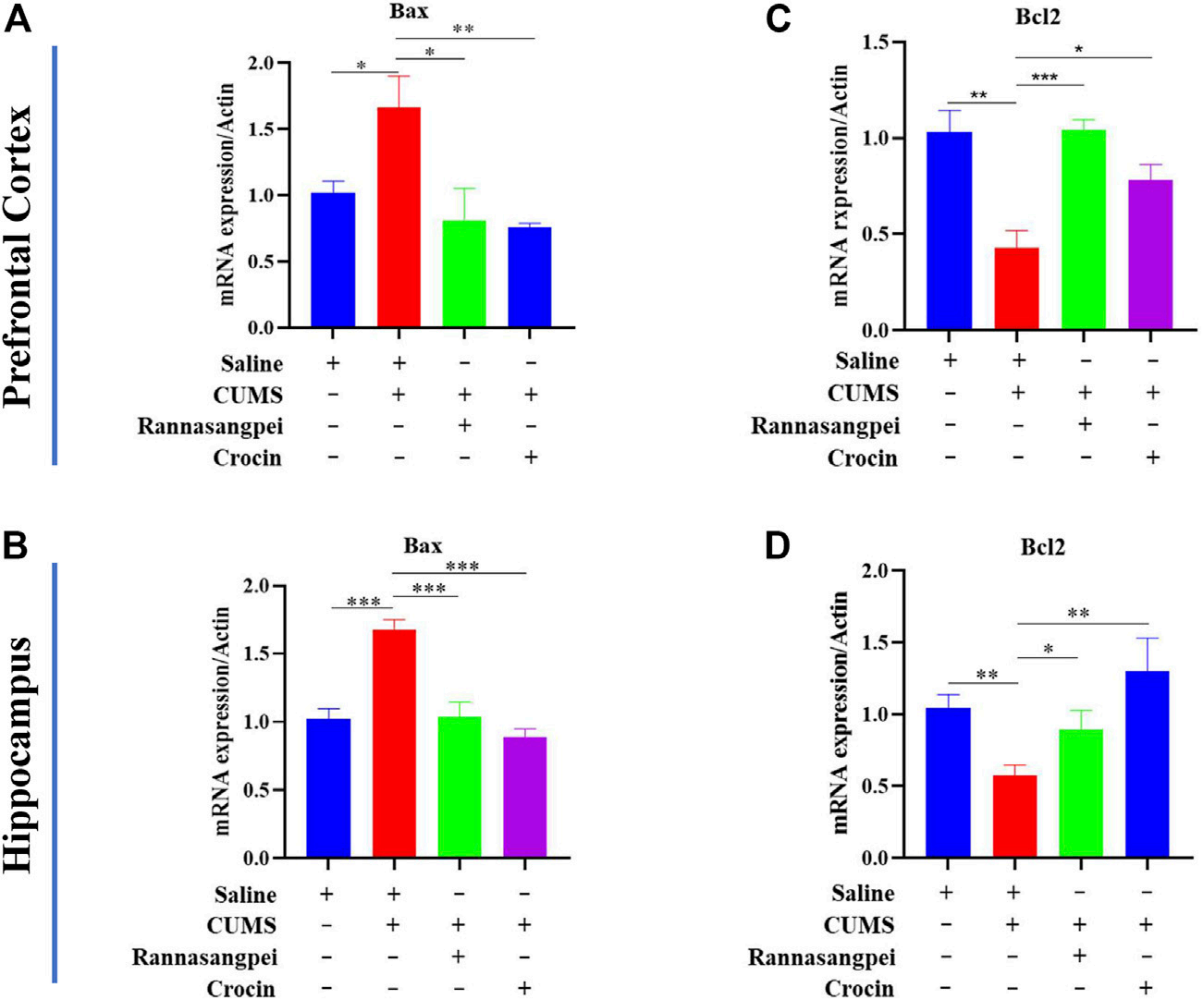
RSNP and crocin-1 ameliorated stress-induced apoptosis in the brains of mice **(A)** Prefrontal cortical and **(B)** Hippocampal Bax expression levels were significantly upregulated in the CUMS-treated mice, while RSNP and crocin-1 administration abolished this alteration. **(C)** Prefrontal cortical and **(D)** Hippocampal Bcl-2 expression levels were significantly downregulated in the CUMS-treated mice, while RSNP and crocin-1 administration abolished this alteration. It should be noted that all the data were normalized to the saline group. RSNP, rannasangpei; CUMS, chronic unpredictable mild stress. N = 9. For ANOVA statistics: **(A)**, F_(3,36)_ = 4.638, *p* = 0.012; **(B)**, F_(3,36)_ = 12.08, *p* < 0.001; **(C)**, F_(3,36)_ = 18.93, *p* < 0.001; **(D)**, F_(3,36)_ = 4.358, *p* = 0.011; *p < 0.05, **p < 0.01, ***p < 0.001.

For apoptosis-related markers, we measured Bcl-2 (anti-apoptotic gene) and Bax (pro-apoptotic gene) mRNA expression levels. The data suggested that Bax mRNA levels were significantly increased in the prefrontal cortex and hippocampus of CUMS-treated mice relative to the saline-treated mice, while RSNP or crocin-1 administration significantly reduced Bax mRNA levels in the CUMS-treated mice (Figures 5A, B). Consistently, the reduced Bcl-2 mRNA levels found in the prefrontal cortex and hippocampus of CUMS-treated mice were rescued by the treatment of RSNP or crocin-1 (Figures 5C, D). These above results indicated that RSNP and crocin-1 had anti-inflammatory and anti-apoptotic effects in CUMS-treated mice.

### RSNP and crocin-1 increased astrocyte proliferation and BDNF expression in CUMS-treated mice

**FIGURE 6:**
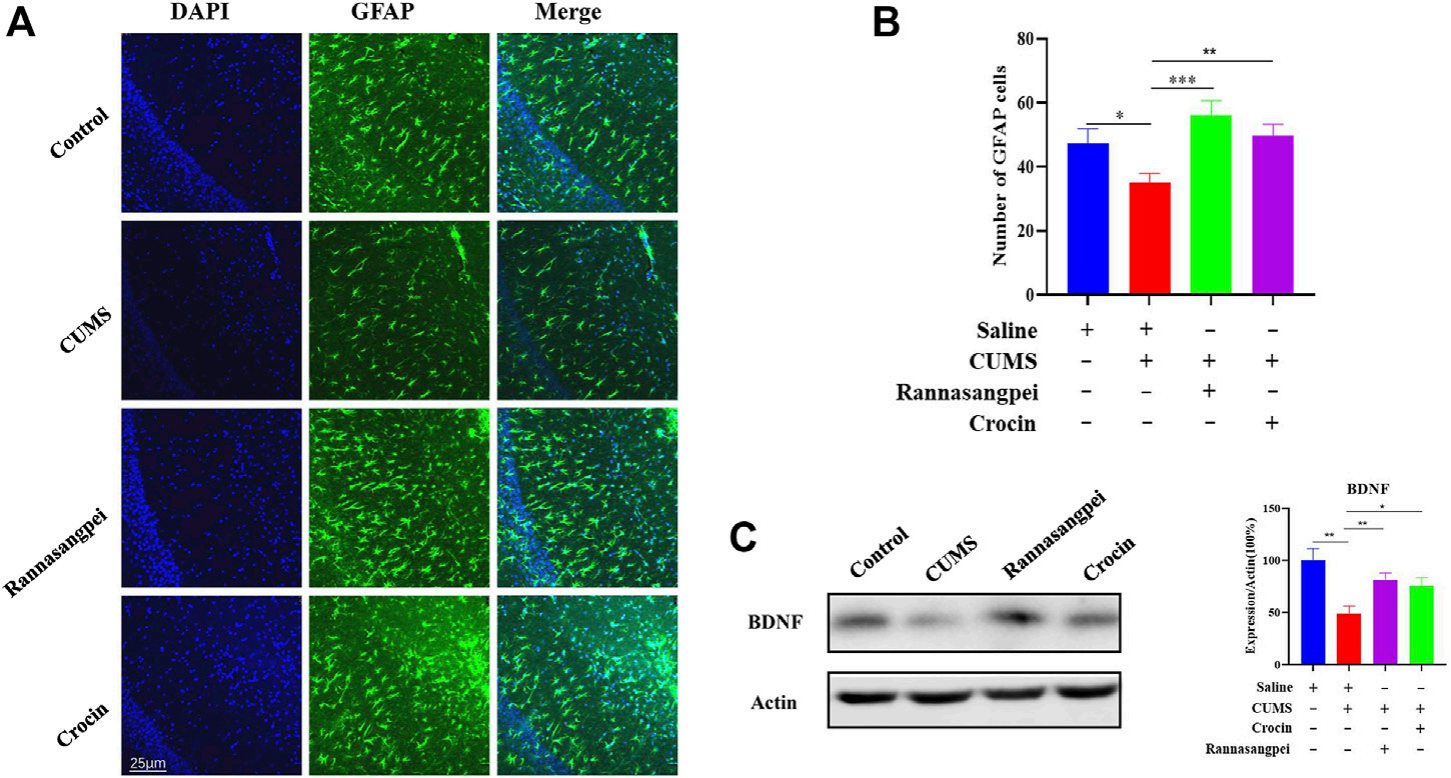
RSNP and crocin-1 increased astrocyte proliferation and BDNF expression in the hippocampus of CUMS-treated mice **(A)** Representative images of GFAP positive cells in the CA1 region of mice under various treatments. **(B)** Quantification of GFAP positive cells in the CA1 region of mice under various treatments. **(C)** RSNP or crocin-1 treatment significantly increased BDNF protein levels in the hippocampus of CUMS model mice. RSNP, rannasangpei; CUMS, chronic unpredictable mild stress; BDNF, brain-derived neurotrophic factor; GFAP, glial fibrillary acidic protein; N = 6. For ANOVA statistics: **(B)**, F_(3,24)_ = 5.610, *p* = 0.0034; **(C)**, F_(3,24)_ = 5.969, *p* = 0.0024. *p < 0.05, **p < 0.01, ***p < 0.001.

We then tested astrocyte number in the mice under various treatments, and the results showed that the astrocyte number in the CA1 region of CUMS-treated mice was decreased (Figures 6A, B), which is consistent with the previous reports (Jiang et al., 2018; Shu et al., 2019). Furthermore, we found that RSNP or crocin-1 administration restored the astrocyte proliferation in the CUMS-treated mice (Figures 6A, B). Consistently, the reduced BDNF protein levels detected in the hippocampus of CUMS-treated mice were partially rescued by RSNP or crocin-1 treatment (Figure 6C), given it has been suggested that BDNF prevented astrocyte death resulting in neuroprotection (Saba et al., 2018). These results indicated that RSNP and crocin-1 increased astrocyte proliferation and BDNF expression in the hippocampus of CUMS-treated mice.

## Discussion

The development of antidepressant drugs over the past few decades has been limited by the pathological complexity of depression. In the present study, however, an effective antidepressant-like effect of RSNP and its active ingredient crocin-1 in a mouse model of depression was validated, and the potential molecular pathways underlying the effects were evaluated by a series of methods. We found that RSNP or crocin-1 exerts its anti-depressant-like effects *via* various molecular pathways reflective of MDD pathophysiology, including reduction of oxidative stress, anti-inflammatory properties, and anti-apoptotic effects. To the best of our knowledge, this research is the first to demonstrate an anti-depressant-like effect of RSNP and/or its active ingredient crocin-1.

It should be noted that there were brain-region specific inflammation-related marker changes in response to chronic stress and/or RSNP (crocin-1) treatment in the mice, and one example was that there were no significant differences among groups evaluating IL-10 mRNA levels in the prefrontal cortex, whereas RSNP or crocin-1 administration significantly restored IL-10 mRNA levels in the hippocampus of CUMS-treated mice. Another example was that the reversal of TNF-α levels induced by crocin-1 was only observed in the hippocampus, but not in the prefrontal cortex. Although it is unclear the reason underlying the brain region-specific changes of TNF-alpha levels induced by crocin-1, a recent study by Naghibi et al. (2021) found that treadmill exercise only reduced anhedonia-like behaviors and hippocampal (but not prefrontal cortex) TNF-α levels in the model rats, which was consistent the data from our present study. Similarly, we observed tissue specific oxidative stress-related marker changes, since MDA levels were modulated in the serum (but not hippocampus) of mice under various treatments. Additionally, the decreased serum SOD activities found in the CUMS-treated mice were significantly rescued by the treatment of RSNP (but not crocin-1), whereas both RSNP and crocin-1 restored hippocampal SOD activities in the model mice. A limitation of this study is that the molecular mechanisms underlying the tissue-region specific inflammation or oxidative stress-related marker changes in response to chronic stress and/or RSNP (crocin-1) treatment in the mice are unclear, and it therefore requires further investigation into this phenomenon. In contrast, the dysregulated Bcl-2 and Bax (apoptosis-related markers) levels in the CUMS-treated mice were rescued by RSNP or crocin-1 both in the prefrontal cortex and hippocampus. Consistently, a study utilized another traditional medicine-electroacupuncture, and also showed that the inhibition of Bcl-2/Bax apoptosis pathway in the hippocampus was involved in electroacupuncture-induced anti-depressant-like effects in CUMS-treated mice (Guo et al., 2021).

Chronic stressful life events are predisposing factors for MDD development, and it is well known that prolonged chronic stress leads to MDD in humans and depressive-like behaviors in animal models (Cheng et al., 2015; Seo et al., 2017). The mice exposed to the CUMS paradigm showed various depressive-like characteristics, such as increased immobility times in the FST and TST, which is a method widely used to successfully establish MDD model and to test the efficacy of new drugs (Yan et al., 2018). Here we showed that the depressive-like behaviors induced by CUMS were reversed by RSNP or crocin-1 treatment, and the changes of these behavioral indices were similar to those induced by the typical antidepressants such as fluoxetine described in the previously published literature (Yan et al., 2020). These results suggested that RSNP and its active components are promising therapeutic target for treatment of MDD, especially considering that the ingredients of RSNP are from natural products and should be of low risks of side effects. In addition, the autonomous motor abilities of MDD mice were similar in the four groups tested in this study, suggesting that the differences in immobility among groups are not due to locomotion deficits.

Although RSNP has been widely used as a Tibetan medicine to treat various acute or chronic diseases such as neurodegenerative diseases for hundreds of years, and listed in the 2015 edition of Pharmacopoeia of China (Nie et al., 2021), the efficacy of this medicine has not been validated by clinical trials and/or preclinical studies for a very long period of time. However, several preclinical studies have been reported to verify the effects of RSNP in recent years. Wu et al. (2016) used a vascular dementia rat model and showed that RSNP improved cognitive impairments in the model rats, and it also rebalanced the oxidative stress status. In another study, Fu et al. (2021) demonstrated that RSNP protected against cerebral ischemia-reperfusion injury by regulating gut microbiota and inhibiting the inflammatory response, and the same group also showed that the beneficial effect of RSNP in cerebral ischemia-reperfusion injury was *via* blood-brain barrier and metabonomics with 18 identified active ingredients (Xu et al., 2020). Furthermore, RSNP improved motor function and ameliorated the pathological lesions in the brains of Parkinson’s disease mice (Hu et al., 2020). Here we for the first time demonstrated a therapeutic role of RSNP in a psychiatric disease from an animal model of MDD, and therefore suggesting that the Tibetan medicine may extend its applications into mental disorders.

One disadvantage of traditional medicine including Tibetan medicine is that the active components mediating the beneficial effects are largely unknown, and therefore limiting their applications. We found that both RSNP and its active ingredient crocin-1 exhibited anti-depressant-like effects, reduced oxidative stress status and anti-inflammatory response in stressed mice. There results were consistent with previous reports on antioxidant and anti-inflammatory properties of crocin-1 (Qiu et al., 2020). Thus, we hypothesized that the antidepressant-like effects of RSNP found in this study were at least partially contributed by crocin-1 through its antioxidant and anti-inflammatory properties. Noticeably, the data from this study indicated that RSNP had higher levels of antioxidant and anti-inflammatory properties than crocin-1 in the CUMS-treated mice, and this is reasonable given that other ingredients in RSNP may contribute to its antioxidant and anti-inflammatory properties. Therefore, our data support the hypothesis that alternative medicine or natural products with two or more compounds are promising to treat acute and chronic diseases, although this requires further validation.

It has long been known that the hippocampal morphology and function were affected by the release of glucocorticoids caused by stress (McEwen, 2000). Glucocorticoid levels fluctuate with menstrual cycles, which might lead to glucocorticoid receptor resistance and sex hormone secretion, and therefore was suggested to be associated with the pathogenesis of female patients with MDD (Zhu et al., 2021). Stress-induced secretion of glucocorticoids has been shown to decrease serotonin, and this may lead to the decreased secretion of neurotrophic factors such as BDNF (Cheng et al., 2015). In fact, clinical studies have demonstrated peripheral blood BDNF levels were downregulated in patients with MDD and it is a potential biomarker to inform the treatment response (Molendijk et al., 2014; Peng et al., 2018). Preclinical studies also revealed BDNF downregulation in the brains of MDD model mice, and BDNF played a critical role in the onset and/or development of depression (Moshe et al., 2016; Zhang et al., 2019). Therefore, it is very likely that the observed BDNF upregulation in the hippocampus of CUMS-treated mice after RSNP or crocin-1 administration partially contributed to the drug’s anti-depressant effects. Additionally, it has been reported that glucocorticoids promotes apoptosis in hippocampus (Duman et al., 1999), and imaging studies have revealed that the volume of hippocampus in MDD patients were smaller than those in healthy volunteers (Kempton et al., 2011). Interestingly, antidepressants were found to increase neural progenitor cells in the human hippocampus (Boldrini et al., 2009) and the reduced hippocampal gray matter was largely restored by antidepressants in the patients (Arnone et al., 2013). Therefore, it is reasonable to found that RSNP or crocin-1 increased hippocampal astrocyte number and reduced apoptosis in the hippocampus of CUMS-treated mice. Based on these observations, we hypothesized that RSNP and its active ingredients may act through different pathways to exert neuroprotective and/or anti-depressant-like effects, and one pathway was through BDNF regulation of cell proliferation and/or apoptosis. However, one limitation of study is that we did not investigate the potential of anti-depressant-like effects of RSNP or crocin-1 in female mice, and sex should be taken into consideration for future studies exploring novel therapeutic agents in MDD. Another limitation of this study is that the toxicology of RSNP is still largely unknown. It is well known that Zuotai, a Tibetan medicine mixture containing β-HgS, has been included in various Tibetan medicines, including RSNP. Liu et al. (2019) suggested that the therapeutic effects and toxicity in Tibetan medicines were largely contributed by chemical compositions of minerals (metals), and RSNP was such an example. They further demonstrated that Zuotai and Zuotai-containing 70W at clinical doses had minimal influence on hepatic mRNA expression of Cyp1a2, Cyp2b10, Cyp3a11, Cyp4a10 and Cyp7a1, and corresponding nuclear receptors in mice, while HgCl 2 and MeHg produced significant effects, suggesting that it is inappropriate to assess the safety of HgS-containing RSNP *via* total Hg content (Nie et al., 2021). Therefore, future well-designed preclinical and clinical studies are encouraged to elucidate the toxicology of Tibetan medicines.

In conclusion, our results were the first to provide evidence that the Tibetan medicine RSNP and its active ingredient crocin-1 had anti-depressant-like effects in an animal model of depression, and the effects were associated with restoration of oxidative stress status, immune system response and apoptotic pathway. Furthermore, RSNP had more potent antioxidant and anti-inflammatory properties that crocin-1 in the stressed mice. Therefore, RSNP and its active ingredients are promising targets to be developed as antidepressants, and future investigations into this translation are warranted, these include the toxicology of RSNP and its active ingredients.

## Data Availability

The raw data supporting the conclusion of this article will be made available by the authors, without undue reservation.
